# Inhalation Devices

**DOI:** 10.1155/2018/5642074

**Published:** 2018-09-18

**Authors:** Petra Moroni-Zentgraf, Omar S. Usmani, David M. G. Halpin

**Affiliations:** ^1^Medical Director ANZ, Boehringer Ingelheim Pty Limited, 78 Waterloo Rd., North Ryde, NSW 2113, Australia; ^2^Reader in Respiratory Medicine & Consultant Physician, National Heart and Lung Institute (NHLI), Imperial College London & Royal Brompton Hospital, Airways Disease Section, Dovehouse Street, London SW3 6LY, UK; ^3^Consultant Physician & Honorary Associate Professor, Royal Devon & Exeter Hospital, Barrack Road, Exeter EX2 5DW, UK

The global burden of chronic respiratory diseases such as asthma and chronic obstructive pulmonary disease (COPD) remains substantial despite available treatments and management guidelines. In this special issue, dedicated to inhalation devices in respiratory disease, the advantages of the inhalation route, the choice of device, and the importance of educating patients in the correct inhalation technique are examined. The use of asthma medication by recreational and elite athletes is also reviewed.

Inhaled medication is the mainstay of treatment for the management of these diseases, conferring a number of advantages, such as direct delivery of the therapeutic drug to the lung in high concentrations, a rapid onset of action, and minimal systemic side effects. However, as detailed in the review by J. M. Borghardt et al., these benefits can only be achieved if drug design considers the properties of inhaled drugs, the device and formulation characteristics, and the influence of patient characteristics. An understanding of the lung and all its associated kinetic processes is necessary to overcome the complex challenges of the inhalational route of administration.

The importance of matching the inhaler device to the patient is expanded in the review by A. Kaplan and D. Price, who discuss the role of the primary care physician in involving the patient in the selection of an appropriate inhaler. This includes taking into account patient-related factors such as age, dexterity, and cognitive ability, as well as considering patient preferences and perceptions regarding inhaler choice. Having chosen the appropriate device in consultation with their patient, it is vital that the primary care physician educates and trains the patient in its correct use, as well as regularly reviews their technique, in order to minimize common patient errors in inhalation and ensure effective use of inhaler devices among their patients. Treating infants and very young children with asthma can be challenging, as the patients are unable to coordinate an inspiratory maneuver with an actuation of an inhaler. A more suitable method of aerosol delivery is via spacers through a facemask. C. Kofman and A. Teper investigated the efficacy of the steroid fluticasone propionate when delivered via nonvalved spacers (NVS) with MDI as compared to valved holding chambers (VHC) with MDI in a small study of children with asthma aged 6 to 20 months. They conclude that long-term treatment with inhaled steroids is more effective when administered by MDI and VHC in infants with asthma.

All touchpoints between patients and healthcare professionals should be optimized to ensure support is provided to the patient. Given that nurse practitioners, both specialist and nonspecialist, are often a primary point of contact for patients with asthma and COPD, they can be responsible for the ongoing evaluation of asthma control, COPD disease progression, and treatment success. Thus, they are in a key position to recognize poor disease control, provide patient education on topics such as inhaler technique, and encourage treatment adherence, all whilst considering patients' views and preferences. J. Scullion presents the nurse practitioner's perspective and describes the nursing cornerstones of inhaler education—“Know it,” “Show it,” “Teach it,” and “Review it.” Linked to the reinforcement of inhaler technique that nurses can provide is the additional consideration of the pharmacist's role in this regard. M. Toumas-Shehata et al. studied inhaler device technique education maintenance over time in pharmacy undergraduates. They demonstrate the importance of repeated training and the consolidation of skills through the training of others and experience with instructing patients in inhaler use.

The needs of patients with respiratory failure also need consideration. Stopping noninvasive ventilation (NIV) in order to administer the corticosteroids and bronchodilators required to treat their condition via inhalers or nebulizers may result in a rapid deterioration of the patient's condition. P. Rzepka-Wrona et al. review the current literature describing small studies where recommendations for nebulization techniques and mask types are given for aerosol therapy coupled with NIV in this special patient population. However, they identify that there remains a need for more precise data coming from large prospective and well-planned real-life studies on nebulization techniques in patients receiving NIV.

Real-life studies are also important to identify different groups of patients with asthma and different asthma phenotypes. Asthma is known to be prevalent in elite endurance athletes, but little is known of its prevalence in competitive recreational athletes. Analysis of the responses to an online survey sent to Swedish endurance athletes indicates that self-reported asthma appears to be higher in this population than in the general Swedish population. This study by A. Näsman et al. of 10,076 athletes concludes that there appears to be an association between high physical activity and self-reported asthma, with a large proportion of recreational athletes using asthma medications regularly. H. Persson et al. report a study in Swedish elite athletes with asthma, where more than three-quarters of those that responded to a postal questionnaire reported using asthma medication, and about one-third reported using bronchodilators and/or inhaled corticosteroids daily. This suggests that these athletes may have uncontrolled asthma and, as with all asthma patients, should be monitored regularly.

This special issue, about inhalation in respiratory diseases, brings into focus the importance of correct inhaler choice, patient education, and the role played by primary care practitioners, nurses, and pharmacists in reinforcing patient education and inhaler technique. It also raises some interesting questions of how to ensure all patients receive the necessary training from healthcare contacts and how specific subsets of respiratory disease treatment require more attention, from treating the youngest patients, to those requiring NIV, through to elite athletes.

